# Sustainable Synthesis of Silver Nanoparticles (Chemical vs Biological) and Their Antimicrobial Activity against Clinical Pathogens

**DOI:** 10.1021/acsomega.5c05917

**Published:** 2025-09-24

**Authors:** Flávia Satie Noguti, Matheus Felipe Celestino, Lucas Teixeira Mori, Jaqueline Portes Pagnoncelli Martins, Giani Andrea Linde, Nelson Barros Colauto, Cleverson Busso, Renato Eising

**Affiliations:** † 441034 Universidade Tecnológica Federal do Paraná (UTFPR), Graduate Program in Bioscience Technologies, Toledo, Paraná 85902-490, Brazil; ‡ Universidade Estadual do Oeste do Paraná (Unioeste), Graduate Program in Chemistry, Toledo, Paraná 85903-220, Brazil; § 67895 Centro Universitário UNIFATECIE, Paranavaí, Paraná 87720-140, Brazil

## Abstract

Silver nanoparticles synthesized via fungal-mediated processes represent an innovative and environmentally sustainable alternative to conventional chemical methods, reducing toxicity and ecological risks without compromising their antimicrobial activity, particularly against multidrug-resistant bacteria. This study aimed to optimize the synthesis of silver nanoparticles using chemical and biological approaches, characterize their physicochemical properties, and evaluate their antibacterial efficacy. Chemical synthesis employed sodium borohydride and carboxymethylcellulose, optimized via factorial design, with optimal conditions determined as 0.7 g L^–1^ carboxymethylcellulose, 6.0 × 10^–3^ mol L^–1^ sodium borohydride, and 3.5 × 10^–4^ mol L^–1^ silver nitrate. Biological synthesis utilized *Aspergillus niger* in a glucose-casein hydrolyzate medium, varying inoculum volume (1 mL, 1.5 mL, and 2 mL). Characterization techniques included UV–vis spectroscopy, total reflection X-ray fluorescence, and transmission electron microscopy. The microdilution assay evaluated antibacterial efficacy against bacterial strains, including ampicillin-resistant *Pseudomonas aeruginosa*. Results show that chemically synthesized silver nanoparticles are smaller, more uniform, and consistently spherical than biologically synthesized nanoparticles. Biologically synthesized nanoparticles also display predominantly spherical morphology, with similar average diameters across different inoculum volumes, although lower inoculum concentrations tend to yield smaller particles. Nanoparticles produced through chemical and biological methods exhibit bacteriostatic and bactericidal properties; however, those synthesized chemically consistently outperform their biological counterparts against all bacterial strains tested. Both nanoparticle types demonstrate significant efficacy against antibiotic-resistant bacteria, reinforcing their potential as alternative antimicrobial agents. These findings emphasize the value of biological synthesis approaches for producing sustainable nanomaterials and offer a viable strategy to address the global health threat of drug-resistant pathogens.

## Graphical abstract

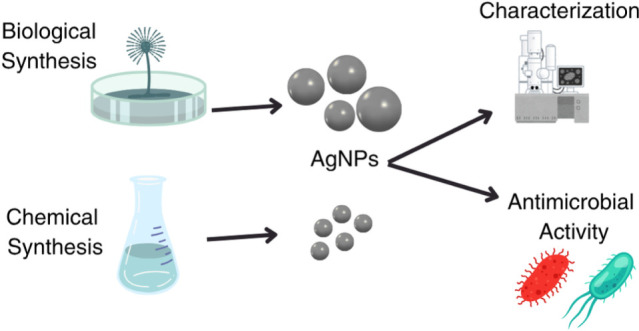

## 1 Introduction

Silver nanoparticles have emerged as a key nanomaterial in biomedical, food safety, and environmental applications due to their potent and broad-spectrum antimicrobial properties.^1^ Unlike bulk silver, silver nanoparticles exhibit enhanced efficacy through their high surface area-to-volume ratio, enabling a sustained release of silver ions that induce oxidative stress and disrupt microbial cell structures.^2^ These nanoparticles exhibit low cytotoxicity, limited potential for resistance development, and efficacy against bacteria^1^—characteristics that position them as a compelling alternative to conventional antimicrobial agents.

The synthesis method plays a decisive role in defining the physicochemical and biological properties of silver nanoparticles. Among chemical routes, the use of strong reducing agents such as sodium borohydride, citrate, or hydrazine remains the most established. In particular, sodium borohydride reduction is widely applied because it enables rapid nucleation, high monodispersity, and reproducibility.^3^ These chemical approaches provide precise control over particle size, morphology, and dispersity, enabling uniform and reproducible nanoparticles.^3^ Further refinement can be achieved through statistical optimization tools, such as factorial design, which allow nanoparticle attributes to be fine-tuned by analyzing interactions among synthesis parameters.^4^ Nonetheless, reliance on hazardous reducing agents raises serious concerns regarding environmental toxicity and biosafety, ultimately limiting the scalability and biomedical applicability of conventional chemical synthesis methods.^5^

In parallel, biological or “green” synthesis has emerged as an eco-friendly and cost-effective alternative. Biological systems such as fungi, bacteria, algae, and particularly plant extracts serve as natural sources of reducing and capping agents, thereby replacing toxic chemicals used in conventional synthesis. Fungal-mediated synthesis benefits from enzymatic activity (e.g., nitrate reductase), while plant-based approaches rely on phytochemicals such as flavonoids and terpenoids that facilitate both reduction and stabilization of nanoparticles.^6^ These methods typically produce nanoparticles coated with biocompatible organic molecules, contributing to reduced ecological impact.^7^ Nonetheless, challenges persist, including heterogeneity in particle size, limited morphological control, and batch-to-batch variability compared with chemical synthesis.^8^ Within biological strategies, fungal-mediated routes—particularly those employing *Aspergillus niger*—offer distinct advantages such as high tolerance to metal ions, efficient extracellular enzyme production, and scalability for industrial applications.^9,10^ The activity of nitrate reductase in *A. niger* facilitates the bioreduction of silver ions, yielding nanoparticles with enhanced stability and reduced environmental impact.^11,12^ Combined with its ubiquity, rapid growth, and high bioaccumulation potential, *A. niger* represents a strategic platform for sustainable nanoparticle synthesis.

Since synthesis routes determine nanoparticle size, morphology, and surface chemistry, they also shape biological activity. A key advance has been the recognition that silver nanoparticles act through multimodal mechanisms—membrane disruption, oxidative stress, and interference with nucleic acids and proteins—unlike antibiotics with single molecular targets. This broad action reduces the risk of resistance and has shown efficacy against multidrug-resistant pathogens such as *Pseudomonas aeruginosa* and *Staphylococcus aureus*.^13,14^

To fully exploit the antimicrobial potential of silver nanoparticles, a detailed characterization of their size, shape, and surface features is essential. Techniques such as UV–visible spectroscopy, transmission electron microscopy (TEM), dynamic light scattering (DLS), and X-ray diffraction (XRD) provide critical insights into these attributes.^5^ These structural and physicochemical characteristics directly affect interactions, including antimicrobial efficacy.

A reliable assessment of antibacterial activity requires robust and quantitative methodologies, which enable precise measurement of antimicrobial efficacy and ensure reproducibility across studies. Such standardized approaches provide a consistent framework for evaluating the performance of silver nanoparticles against clinically relevant pathogens.^15–17^ While qualitative assays such as disk diffusion provide preliminary insights, quantitative methods like broth microdilution provide minimum inhibitory concentration (MIC) and minimum bactericidal concentration (MBC) values—key metrics for evaluating antimicrobial strength.^18,19^ It is also essential to assess efficacy against a representative panel of bacteria. Gram-positive species such as *Clostridium perfringens* and *S. aureus* are notorious for causing foodborne illnesses and antibiotic-resistant infections,^20,21^ whereas Gram-negative pathogens like *Escherichia coli* and *P. aeruginosa* are associated with severe nosocomial and enteric infections.^22,23^

Given the rising threat of antimicrobial resistance and the limitations of current therapies, there is an urgent need for innovative, safe, and sustainable antimicrobial agents. Green-synthesized silver nanoparticles, particularly those produced using *A. niger*, hold promise as next-generation materials in combating multidrug-resistant pathogens. Therefore, this study aimed to optimize the synthesis of silver nanoparticles using chemical and biological (fungal-mediated) methods, characterize their physicochemical properties, and evaluate their antibacterial activity against clinically relevant bacteria. The findings are expected to contribute to developing effective antimicrobial strategies with potential applications in healthcare, food preservation, and environmental protection.

## 2 Materials and Methods

### 2.1 Materials

All reagents employed in this study were of analytical grade (PA) and used without further purification. The chemicals included carboxymethylcellulose (CMC, Sigma-Aldrich, 99%, Mw 700,000), silver nitrate (AgNO_3_, Proquimios, 99%), sodium borohydride (NaBH_4_, Sigma-Aldrich, 98%), polysorbate-20 (Proquimios), and resazurin (7-hydroxy-10-oxidophenoxazin-10-ium-3-one, Sigma-Aldrich). All aqueous solutions were prepared using ultrapure water (resistivity ≥ 18.2 MΩ cm), which was previously degassed by heating to eliminate dissolved gases. Glassware used for solution preparation and nanoparticle synthesis was initially soaked in concentrated nitric acid, followed by thorough rinsing with ultrapure water to avoid contamination.

Microbiological culture media included potato dextrose agar (PDA, HIMEDIA) and cation-adjusted Mueller-Hinton broth (Sigma-Aldrich). Additional reagents used for bacterial growth and viability assays were saline solution (0.9% NaCl, Laborasa-LBS), glucose (Vetec, 99.5%), hydrolyzed casein (Sigma-Aldrich, 96%), polysorbate-80 (Proquimios), and ampicillin (Sigma-Aldrich). All glassware for microbial experiments was washed, rinsed with ultrapure water, and autoclaved at 121 °C for 15 min. Sterile disposable Petri dishes (90 mm) and 96-well microdilution plates were used in all microbiological assays.

Microorganisms used in the study were sourced from the Culture Collection of the National Institute for Quality Control in Health (INCQS), Oswaldo Cruz Foundation (FIOCRUZ), Rio de Janeiro, Brazil. The bacterial strains included: *Clostridium perfringens* (Veillon and Zuber) Hauduroy et al. (INCQS 00053), *Escherichia coli* (Migula) Castellani and Chalmers (INCQS 00033), *Pseudomonas aeruginosa* (Schroeter) Migula (INCQS 00025), and *Staphylococcus aureus* subsp. *aureus* Rosenbach (INCQS 00015). The fungal strain used for biological nanoparticle synthesis was *Aspergillus niger* van Tieghem (INCQS 40371).

### 2.2 Methods

#### 2.2.1 Factorial Design for the Chemosynthesis of Silver Nanoparticles

A factorial design was employed with two key independent variables: carboxymethylcellulose and sodium borohydride concentrations to optimize the chemical synthesis of silver nanoparticles. These parameters were selected due to their critical roles in nanoparticle formation and stabilization. CMC acts as a capping and stabilizing agent, affecting nanoparticle dispersion and growth, while sodium borohydride is a potent reducing agent, initiating rapid nucleation and formation of silver nanoparticles.

A two-level full factorial design was implemented, incorporating four central and four axial (star) points, culminating in 12 experimental runs. This design enabled the assessment of linear, interaction, and quadratic effects between the two variables. The concentration of silver nitrate was held constant at 0.35 × 10^–3^ mol L^–1^ across all experiments to ensure consistent availability of silver ions for reduction. The specific levels (low, high, central, and axial) for each factor are detailed in Table 1, which outlines the experimental matrix used for statistical analysis and response optimization. Each factorial condition listed in Table 1 was tested independently under the standard synthesis protocol, and all experiments were conducted in duplicate to ensure reproducibility.

**Table 1:** Factorial Design Matrix with Concentration Levels of Carboxymethylcellulose (CMC) and Sodium Borohydride (NaBH_4_) for the Chemical Synthesis of Silver Nanoparticles^a^

	Final concentration	
Variable level	CMC (g L^–1^)	NaBH_4_ (mol L^–1^)
**+2**	1.000	10.000 × 10^–3^
**+1**	0.775	7.625 × 10^–3^
**0**	0.550	5.250 × 10^–3^
**–1**	0.280	2.875 × 10^–3^
**–2**	0.100	0.500 × 10^–3^

a Silver nitrate was maintained at a constant concentration of 0.35 × 10^–3^ mol·L^–1^.

The concentration range for sodium borohydride was chosen to maintain a minimum [sodium borohydride]/[silver nitrate] molar ratio of 1.4. Preliminary laboratory assays demonstrated that ratios below this threshold yielded negligible analytical responses. However, ratios exceeding 28.6 led to vigorous hydrogen gas evolution complications due to the rapid decomposition of sodium borohydride in aqueous solution. In parallel, carboxymethylcellulose concentrations were varied between 0.100 and 1.000 g L^–1^ to assess its role as a stabilizing agent, affecting the formation and growth of silver nanoparticles.

All solutions were prepared using degassed ultrapure water (resistivity ≥ 18.2 MΩ cm). Carboxymethylcellulose solutions were stirred overnight to ensure complete dissolution and homogeneity. Sodium borohydride was freshly prepared in ice-cold ultrapure water to minimize decomposition and control hydrogen gas release.

Each synthesis reaction was performed in a 2.0 mL total volume, as follows: 1.8 mL of aqueous carboxymethylcellulose solution was mixed with 0.1 mL of silver nitrate (0.35 × 10^–3^ mol L^–1^) and incubated for 10 min at room temperature. Subsequently, 0.1 mL of freshly prepared sodium borohydride solution was added. All reactions were conducted under light-protected conditions to prevent photoreduction, and the mixtures were manually homogenized. Syntheses were performed in duplicate to ensure reproducibility. UV–vis spectrophotometric analysis was conducted 2 h after sodium borohydride addition.

The analytical response was assessed by scanning each sample in a quartz cuvette using a UV–vis absorption spectrophotometer (ThermoScientific Genesys 10-S), operating in the wavelength range of 300 to 800 nm. During this analysis, three spectral parameters were recorded: the maximum absorbance (Amax), which is associated with the yield of silver nanoparticles; the wavelength at which this maximum absorbance occurs (λmax), which reflects the average particle size; and the full width at half-maximum (fwhh), which indicates the degree of size dispersion within the sample.

To integrate these spectral parameters into a single metric of synthesis efficiency and nanoparticle quality, the analytical response (ψ) was calculated using eq 1:

1 ψ=Amax/(λmax⁡×FWHH)

Where:

ψ = analytical response

Amax = maximum absorbance

λmax = wavelength of maximum absorbance

fwhh = full width at half-height

A higher analytical response (ψ) is associated with greater nanoparticle yield (higher Amax), smaller average particle size (lower λmax), and narrower size distribution (lower fwhh), reflecting the formation of small, monodisperse nanoparticles. The calculated ψ values were incorporated into the factorial design and analyzed using Statistica 13.3 software (StatSoft South America, Quest Software Inc., OK, USA; Serial No. JPZ711I235230FA-T) to generate a response surface model and optimize synthesis conditions.

#### 2.2.2 Chemosynthesis of Silver Nanoparticles Based on Factorial Design

Following the optimization of reagent concentrations using response surface methodology, a 200 mL batch of silver nanoparticle dispersion was synthesized under the established optimal conditions. First, 180 mL of an aqueous carboxymethylcellulose solution (0.78 g L^–1^) was placed in a 500 mL Erlenmeyer flask, to which 10 mL of silver nitrate solution (7.0 × 10^–3^ mol L^–1^) was added. The mixture was homogenized and incubated for 10 min at room temperature (25 °C) without agitation, protected from light with aluminum foil. Subsequently, 10 mL of freshly prepared aqueous sodium borohydride solution (1.2 × 10^–1^ mol L^–1^) was added, and the suspension was homogenized by manual stirring. All reactions were carried out under light-protected conditions to prevent photoreduction, and syntheses were performed in duplicate to ensure reproducibility. Analyses and further experiments were initiated only after 2 h of sodium borohydride addition. The resulting colloidal suspension was lyophilized at −40 °C and 10^–3^ mbar to preserve nanoparticle integrity. To ensure complete drying, an additional portion of the chemosynthesized silver nanoparticles (CSN) was dried in a forced-air oven at 50 °C for 24 h and subsequently stored in a desiccator at room temperature (25 °C) under vacuum until further use.

#### 2.2.3 Biosynthesis of Silver Nanoparticles Using Fungal Suspension Filtrate

The fungal strain *A. niger* was initially cultured on PDA (39 g L^–1^) at 27 ± 1 °C for approximately 7 days or until complete surface colonization of 90 mm Petri dishes. After incubation, 20 mL sterile aqueous saline solution (0.9% NaCl) containing 0.02 mL polysorbate-20 was added over the fungal biomass. Spores were gently dislodged from the fungal biomass using a Drigalsky loop, and the resulting spore suspension was collected. The spore concentration was determined in duplicate using a Neubauer counting chamber to standardize inoculum volumes.

To prepare the fungal biomass, 1.0, 1.5, and 2.0 mL aliquots of the spore suspension (6.4 × 10^6^ spores per mL) were used as inocula in 250 mL Erlenmeyer flasks containing 100 mL glucose–casein hydrolyzate liquid medium. The medium was prepared by dissolving 4 g casein hydrolyzate in 500 mL of distilled water and 5 g glucose in 500 mL of distilled water; both solutions were autoclaved at 121 °C for 15 min and then mixed. The flasks were incubated on an orbital shaker (Thoth Equipment, Series 23140) at 27 ± 1 °C and 150 rpm for 72 h. To prevent contamination, all procedures were carried out under aseptic conditions and in triplicate.

Following the incubation period, the mycelial biomass was separated from the culture broth using a 200-mesh sieve and transferred to a fresh 250 mL Erlenmeyer flask containing 100 mL autoclaved distilled water (121 °C for 15 min). This washing step was included to remove residual culture medium and to promote the release of extracellular metabolites, such as reductive enzymes and proteins, into the aqueous phase, thereby providing the active components required for nanoparticle synthesis.^10^ The suspension was incubated for an additional 48 h under the same conditions (27 ± 1 °C, 150 rpm). After incubation, the fungal suspension was filtered through Whatman No. 1 filter paper to obtain the cell-free filtrate, which was immediately used for silver nanoparticle biosynthesis. The remaining fungal biomass was discarded.

To initiate biosynthesis, 50 mL fungal suspension filtrate was mixed with 10 mL silver nitrate solution (10 mmol L^–1^) and incubated at 27 ± 1 °C for 24 h in an orbital incubator under static conditions, with the glassware protected from light using aluminum foil. The formation of silver nanoparticles was confirmed by a visible color change in the reaction mixture, from cloudy white to brownish hues, consistent with that reported by Moharekar et al.^24^

The biosynthesized silver nanoparticles (BSN) were categorized based on the volume of spore suspension used in the initial liquid culture. Specifically, BSN1 refers to nanoparticles synthesized with 1.0 mL of spore inoculum, BSN2 with 1.5 mL, and BSN3 with 2.0 mL. This coding system was adopted to facilitate clear identification of synthesis conditions and ensure reproducibility across batches. Each synthesis condition (BSN1–3) was performed in triplicate with independent fungal cultures to ensure batch-to-batch consistency. After biosynthesis, the dispersions were centrifuged twice at 200 × *g* for 20 min in 30 mL Falcon tubes, without subsequent washing of the pellets. The resulting supernatant, containing the BSN dispersions, was stored at 4 °C and used directly for characterization and antibacterial assays without further purification. Before use, samples were gently vortexed to ensure homogeneity.

#### 2.2.4 Characterization of Silver Nanoparticles

The characterization of silver nanoparticles, chemosynthesized (CSN) and biosynthesized (BSN1, BSN2, and BSN3), was carried out using a combination of spectroscopic and microscopic techniques to evaluate their optical, elemental, and morphological properties.

The optical behavior of the nanoparticles was assessed via UV–visible absorption spectroscopy ThermoScientific Genesys 10-S spectrophotometer. Spectra were recorded in the wavelength range of 300 to 800 nm, employing quartz cuvettes and using distilled water as the reference blank in all measurements.

Elemental analysis of the biosynthesized silver nanoparticles (BSN1, BSN2, and BSN3) and the CSN was performed using total reflection X-ray fluorescence (TXRF) spectroscopy with a Bruker S2 PICOFOX spectrometer (Bruker AXS Microanalysis GmbH), following the methodology described by Ribeiro et al.^25^ For each analysis, 2 mL Eppendorf tubes containing 900 μL of the nanoparticle sample and a gallium standard solution (CGa = 100 mg L^–1^) were prepared. A 5 μL aliquot of each prepared mixture was deposited at the center of a quartz disk and left to dry under ambient conditions in a laminar flow cabinet.

Transmission electron microscopy (TEM) was used to evaluate the morphology and size distribution of the synthesized nanoparticles. TEM images were acquired using a JEM-1011 microscope operated at an acceleration voltage of 100 kV. For sample preparation, a drop of nanoparticle dispersion was deposited onto a 200-mesh copper grid coated with a carbon film, and excess liquid was removed with filter paper to prevent particle aggregation. The grid was dried at room temperature (25 °C) in a desiccator. Nanoparticle diameters were measured using ImageJ software, based on a minimum of 150 particles per sample. Measurements were performed on at least 150 particles per sample, collected from multiple regions of the TEM grid to ensure representative analysis. These measurements were used to determine the average diameter and assess the size distribution of the nanoparticles.

#### 2.2.5 Antibacterial Activity Assay

The antibacterial activity of chemosynthesized (CSN) and biosynthesized (BSN1, BSN2, BSN3) silver nanoparticles was evaluated using the broth microdilution method, following the standardized protocol established by the Clinical and Laboratory Standards Institute.^18^ Bacterial suspensions were prepared in sterile saline solution and adjusted to a turbidity equivalent to the 0.5 McFarland standard, corresponding to approximately 2 × 10^8^ CFU mL^–1^. Inocula were freshly prepared on the day of use, stored at 4 °C, and validated by serial dilution and plating on solid media to confirm viability and absence of contamination.

The minimum inhibitory concentration (MIC) and minimum bactericidal concentration (MBC) were determined using sterile 96-well microtiter plates. Each well received 100 μL cation-adjusted Mueller-Hinton broth, previously autoclaved at 121 °C for 15 min. Silver nanoparticles were prepared at an initial concentration of 0.4719 μg mL^–1^ by dissolving them in 5 mL of a polysorbate-80 solution (0.1 mL 100 mL^–1^). Subsequently, 100 μL nanoparticle suspension and 100 μL bacterial inoculum (final concentration: 2 × 10^4^ CFU per well) were added to each well. All experiments included sterile negative controls (broth only) and solvent controls to confirm the absence of contamination. Each MIC and MBC assay was independently conducted in triplicate with freshly prepared inocula and nanoparticle suspensions.

As confirmed by optical microscopy, the MIC was defined as the lowest nanoparticle concentration that completely inhibited visible bacterial growth. A colorimetric assay based on resazurin reduction was employed to further verify microbial inhibition. In this method, viable bacteria reduce blue resazurin to pink resorufin, indicating metabolic activity.

To determine the MBC, 2 μL aliquots were transferred from wells showing no visible growth into new wells containing 100 μL fresh cation-adjusted Mueller-Hinton broth. Plates were incubated for 24 h at 35 °C, after which 20 μL resazurin solution was added to each well. The MBC was considered the lowest concentration that showed no color change, corresponding to *a* ≥ 99.5% reduction in viable bacterial cells relative to the initial inoculum.

Optical density was measured at 655 nm using a microplate reader. A blank control (broth medium with diluted silver nanoparticles but no bacteria) and a positive control (untreated bacterial inoculum) were included in all assays. Ampicillin (1 mg mL^–1^ in sterile saline solution) was used as a positive control for antimicrobial activity, while polysorbate-80 (0.1 mL 100 mL^–1^) was a negative control to assess potential solvent effects.

All MIC and MBC determinations were performed in triplicate, using independent bacterial inocula and freshly prepared silver nanoparticle suspensions for each replicate. Reported values correspond to the lowest concentration consistently observed to inhibit (MIC) or eliminate (MBC) bacterial growth across the three replicates, in accordance with Clinical and Laboratory Standards Institute (CLSI) guidelines.^18^ As MIC and MBC are qualitative threshold end points, standard deviations were not reported, and the results were presented as discrete consensus values without error bars.

## 3 Results

### 3.1 Chemosynthesis of Silver Nanoparticles: Optimization via Factorial Design

The spectroscopic parameters obtained from the factorial design for the CSN (Table 1) were used to calculate the analytical response (ψ) and to construct the corresponding response surface (Figure 1). The response variable, which reflects the efficiency of nanoparticle formation, exhibited a pronounced dependence on sodium borohydride and carboxymethylcellulose concentrations.

**Figure 1:**
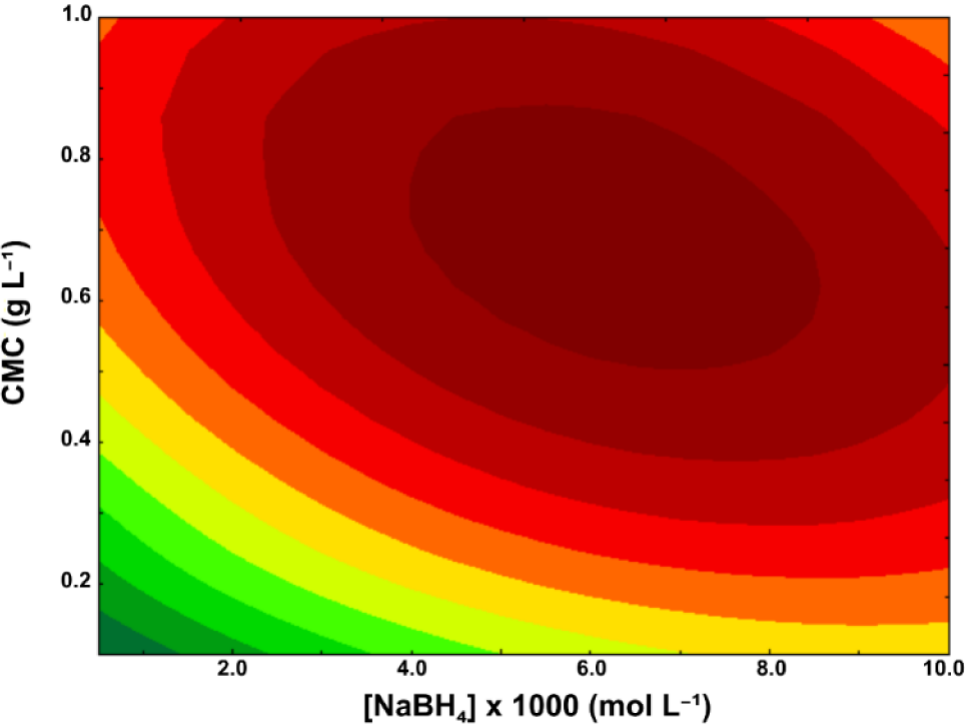
Response surface derived from factorial design showing the effect of sodium borohydride (NaBH_4_) and carboxymethylcellulose (CMC) concentrations on silver nanoparticle chemosynthesis at 3.5 × 10^–4^ mol L^–1^ silver nitrate.

The response surface revealed a progressive increase in the analytical response as sodium borohydride and carboxymethylcellulose concentrations increased, with a clearly defined optimum observed at approximately 6.0 × 10^–3^ mol L^–1^ NaBH_4_ and 0.7 g L^–1^ CMC, under a fixed concentration of 3.5 × 10^–4^ mol L^–1^ silver nitrate. This condition corresponded to the maximum analytical response, indicating favorable conditions for nanoparticle synthesis.

The elliptical shape of the contour lines suggests a statistically significant interaction between the reducing and stabilizing agents. The optimal response could not be achieved by increasing either sodium borohydride or carboxymethylcellulose individually; instead, it required a synergistic balance between both variables. Conversely, suboptimal conditions were observed at lower concentrations, particularly below 2.0 × 10^–3^ mol L^–1^ NaBH_4_ and 0.2 g L^–1^ CMC, where the response values were markedly reduced, likely reflecting inefficient reduction and/or insufficient particle stabilization.

These findings underscore the importance of jointly optimizing sodium borohydride and carboxymethylcellulose concentrations to enhance chemosynthesis performance in silver nanoparticle production under fixed precursor concentration conditions.

### 3.2 Biosynthesis of Silver Nanoparticles: Fungal-Mediated Routes

The spore suspension achieved a concentration of 6.4 × 10^6^ spores per mL. Accordingly, inoculum volumes of 1.0, 1.5, and 2.0 mL provided initial spore counts of 6.4 × 10^6^, 9.6 × 10^6^, and 12.8 × 10^6^ spores, respectively, for treatments BSN1, BSN2, and BSN3. Following inoculation and cultivation, the resulting dry fungal biomass was 8.702 g (BSN1), 9.411 g (BSN2), and 10.331 g (BSN3).

Increasing the inoculum volume led to higher fungal biomass production, though the trend was nonlinear. At the same time, the inoculum increased by 50% from BSN1 to BSN2 and 33% from BSN2 to BSN3; the corresponding fungal biomass increased by only 8.15% and 9.78%, respectively. The calculated inoculum-to-biomass efficiency ratios were 16.3% (BSN1 to BSN2) and 29.3% (BSN2 to BSN3), suggesting diminishing returns in fungal biomass yield with increasing inoculum volume. These results indicate a sublinear growth pattern.

This reduced biomass efficiency may be attributed to limitations in the culture medium, including nutrient depletion, oxygen restriction, spatial constraints, and the accumulation of inhibitory metabolites, which likely limit fungal proliferation under higher inoculum conditions.

All BSN cultures exhibit a characteristic color change from cloudy white to shades of brown, consistent with the formation of silver nanoparticles. Specifically, BSN2 and BSN3 develop more yellowish hues, while BSN1 displays a deeper brown color by the end of cultivation. These chromatic variations reflect differences in silver ions’ reduction and nucleation processes, potentially affecting the synthesized nanoparticles’ formation rate, stability, size, shape, and degree of aggregation.

### 3.3 Characterization of Chemosynthesized and Biosynthesized Silver Nanoparticles

The UV–vis spectrum of the optimized CSN exhibited a well-defined surface plasmon resonance peak at approximately 390 nm (Figure 2A). This peak is characteristic of colloidal silver nanoparticles and is typically associated with spherical particles, which display surface plasmon resonance in the 400–500 nm range.^26^ The unimodal profile and absence of secondary absorption bands indicated minimal aggregation and a narrow size distribution (Figure 2A). In addition, the high maximum absorbance (Amax) and low full width at half-maximum (fwhm) values further supported the uniformity and stability of the nanoparticles. These spectral features validated the effectiveness of the factorial design and confirmed that the selected factor levels (Figure 1) successfully converged at an optimal synthesis point. The position and sharpness of the surface plasmon resonance band reinforce that the synthesized nanoparticles were predominantly spherical and well-dispersed.

**Figure 2:**
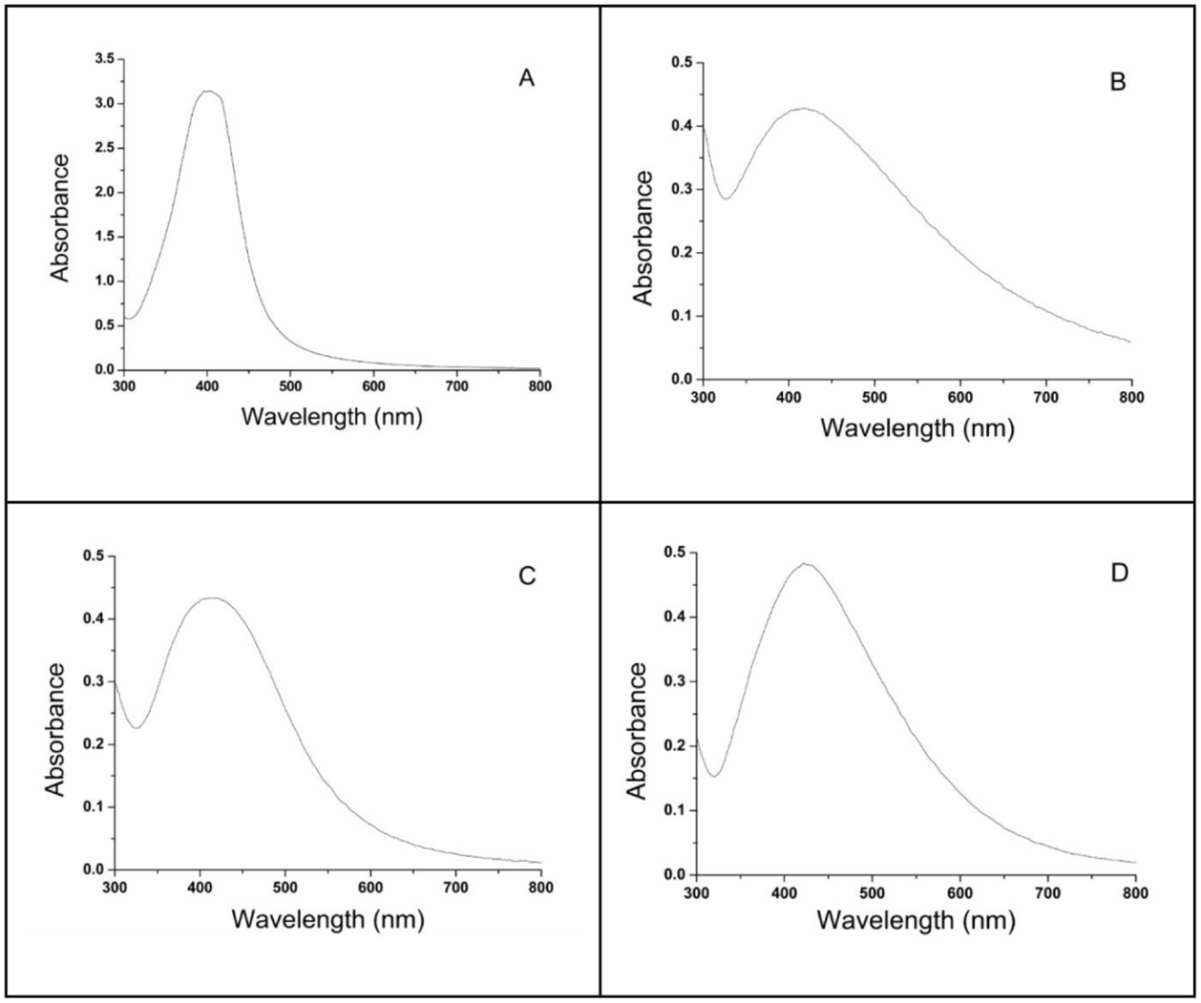
UV–vis spectra of silver nanoparticles obtained by chemosynthesis. (A) CSN under optimal conditions (0.7 g L^–1^ carboxymethylcellulose, 6.0 × 10^–3^ mol L^–1^ sodium borohydride, and 3.5 × 10^–4^ mol L^–1^ silver nitrate) and biosynthesis: (B) BSN1, (C) BSN2, and (D) BSN3, using *Aspergillus niger* spore suspensions of 1 mL (6.4 × 10^6^ spores), 1.5 mL (9.6 × 10^6^ spores), and 2 mL (12.8 × 10^6^ spores), respectively, cultivated in 100 mL glucose-casein hydrolyzate liquid medium.

In contrast to the CSN, the BSN exhibited distinct spectral features that vary with the initial *A. niger* spore concentration. BSN1 (Figure 2B), produced from 1.0 mL spore suspension, showed a broad and less intense surface plasmon resonance band centered around 430 nm, indicative of a lower nanoparticle concentration and greater polydispersity. BSN2 (Figure 2C), generated with 1.5 mL spores, exhibited a sharper surface plasmon resonance peak near 425 nm, reflecting improved nanoparticle formation and more uniform size distribution. BSN3 (Figure 2D), synthesized from 2.0 mL spores, presented the most well-defined and intense surface plasmon resonance band at approximately 420 nm, closely matching the spectral profile of the optimized CSN sample.

Across all BSN treatments, the UV–vis spectra consistently display a primary absorption band between 400 and 500 nm, characteristic of spherical silver nanoparticles (Figure 2B–D). Additionally, a secondary peak near 300 nm is observed in each spectrum, corresponding to fungal proteins involved in nanoparticle reduction and stabilization.^5^ The extended absorption tail between 500 and 800 nm suggested the coexistence of nonspherical morphologies, indicating a heterogeneous mixture of nanoparticle shapes formed during biosynthesis.

The spectral progression from BSN1 to BSN3 demonstrated that increasing fungal biomass enhances the efficiency of silver ion reduction, resulting in greater nanoparticle uniformity and stability. The convergence between BSN3 and CSN spectra further suggests that, under optimized biological conditions, biosynthesis could yield nanoparticles with physicochemical properties comparable to those produced by chemical methods.

Moreover, the unimodal character of the absorption bands in CSN and BSN samples, combined with the absence of secondary peaks and the presence of a symmetric Gaussian profile centered around the surface plasmon resonance maximum, confirms the high quality and monodispersity of the synthesized nanoparticles.^27^ These findings support the effectiveness of both synthesis strategies in producing stable, spherical silver nanoparticles with minimal aggregation.

Transmission electron microscopy (TEM) of CSN under optimized conditions reveals a well-defined and uniform morphology (Figure 3A). The micrograph showed predominantly spherical nanoparticles, well-dispersed with minimal agglomeration, indicating effective steric stabilization by the carboxymethylcellulose matrix (Figure 3A). The narrow size distribution and consistent shape reflect chemically controlled nucleation and growth processes, highlighting the reproducibility of the synthesis route and the precise control over reaction kinetics.

**Figure 3:**
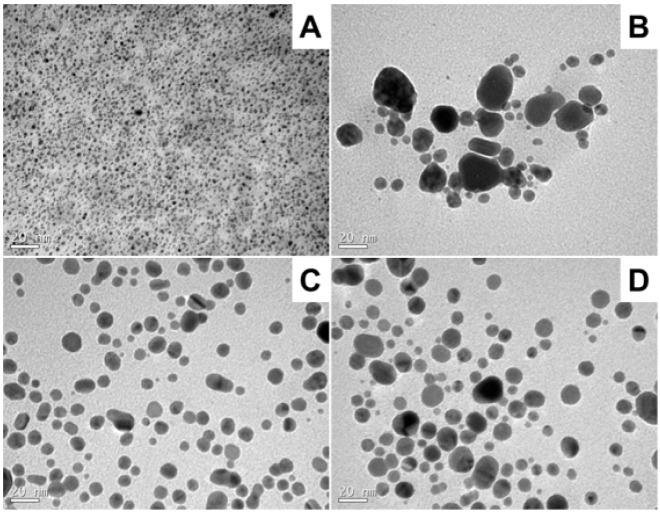
Transmission electron microscopy micrographs of silver nanoparticles synthesized via two approaches: (A) Chemically synthesized nanoparticles (CSN), obtained under optimized conditions (0.7 g L^–1^ carboxymethylcellulose, 6.0 × 10^–3^ mol L^–1^ sodium borohydride, and 3.5 × 10^–4^ mol L^–1^ silver nitrate); and (B–D) Biosynthesized nanoparticles (BSNs), produced using *Aspergillus niger* spore suspensions of (B) 1 mL (6.4 × 10^6^ spores), (C) 1.5 mL (9.6 × 10^6^ spores), and (D) 2 mL (12.8 × 10^6^ spores), each cultivated in 100 mL glucose-casein hydrolyzate liquid medium. The scale bar represents 20 nm.

In contrast, BSNs labeled BSN1, BSN2, and BSN3 displayed greater morphological and dimensional variability (Figure 3B–D). The TEM micrographs revealed three main particle shapes—spherical, triangular, and rod-like—across all BSN samples. However, only spherical nanoparticles were considered for quantitative analysis. BSN1 (Figure 3B) showed a moderate dispersion degree and nanoparticles with slightly irregular edges, encompassing a diverse range of morphologies, particularly spherical shapes. BSN2 (Figure 3C) showed increased particle density and clustering, likely due to higher enzymatic activity from the elevated spore concentration, which also favored the formation of larger spherical nanoparticles. BSN3 (Figure 3D) demonstrated extensive aggregation and reduced morphological uniformity, including larger spherical particles, potentially due to the saturation of biological reducing and stabilizing agents at the highest spore concentration. The BSN samples displayed a broader size distribution and a higher tendency toward agglomeration than CSN. This behavior can be attributed to the complex and less predictable nature of biological reducing agents and the limited stabilization offered by biomolecules in biological synthesis compared to polymers in chemosynthesis. The diversity in particle shapes and sizes further reflects the inherent variability of the biosynthetic process. Overall, the findings indicate that while chemical synthesis produces highly uniform and stable silver nanoparticles, biosynthesis introduces greater heterogeneity in shape and size. Additionally, spore concentration is critical in governing nucleation and growth during biosynthesis, affecting particle uniformity, dispersion, and aggregation behavior.

The size distribution profile of CSN, produced under optimal conditions, exhibited a narrow distribution range, with most particles measuring between 1.5 and 3 nm (Figure 4A). The highest frequency occurred at 2.0 nm, representing 44% of the particles, followed by 2.5 nm at 24.5% (Figure 4A). This narrow size dispersion underscores the high degree of uniformity and control achieved through chemical synthesis.

**Figure 4:**
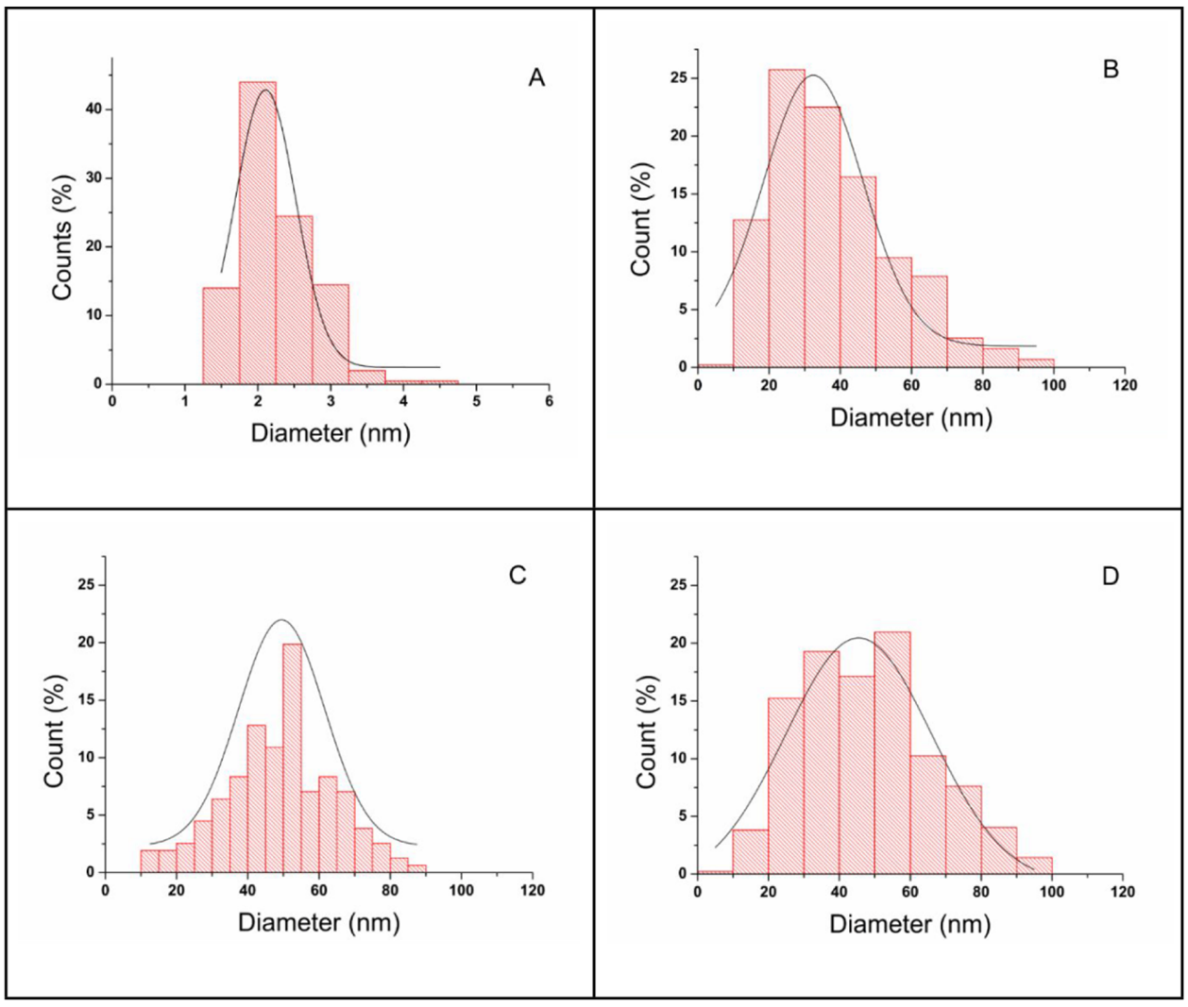
Histograms of silver nanoparticle size distribution obtained by chemosynthesis (A) CSN, using optimal conditions (0.7 g L^–1^ carboxymethylcellulose, 6.0 × 10^–3^ mol L^–1^ sodium borohydride, and 3.5 × 10^–4^ mol L^–1^ silver nitrate); and by biosynthesis using *Aspergillus niger* spore suspensions (B) BSN1 with 1 mL (6.4 × 10^6^ spores), (C) 1.5 mL (9.6 × 10^6^ spores), and (D) 2 mL (12.8 × 10^6^ spores), each cultivated in 100 mL glucose-casein hydrolyzate liquid medium. The distributions follow symmetric Gaussian profiles.

In contrast, the size distributions of BSNs using increasing concentrations of *A. niger* spores are shown in Figure 4B–D. BSN1 (Figure 4B), produced with 1 mL of spore suspension, exhibited a broader size distribution, with particle diameters ranging from 15 to 45 nm and comprising 74.4% of the sample. The highest frequency occurred at 25 nm (25.8%), followed by 35 nm (22.5%) and 45 nm (16.4%). BSN2 (Figure 4C), obtained with 1.5 mL of spores, presented a bimodal distribution, suggesting the coexistence of smaller (<10 nm) and larger (>20 nm) nanoparticles, likely due to heterogeneous nucleation environments. The predominant size range extended from 37.5 to 67.5 nm, accounting for 73.6% of the particles, with the highest frequencies at 52.5 nm (19.9%), 42.5 nm (12.8%), and 47.5 nm (10.9%). BSN3 (Figure 4D), produced with 2 mL spore suspension, displayed the broadest size distribution and a pronounced presence of larger particles. Particle sizes ranged from 25 to 55 nm, accounting for 72.4% of the total, with the highest frequencies at 55 nm (21.0%), 35 nm (19.3%), and 45 nm (17.1%). This pattern indicates secondary growth and aggregation at higher spore concentrations.

The histograms confirm that CSN exhibits significantly greater size uniformity than its biosynthesized counterparts. The progressive increase in spore concentration during biosynthesis resulted in broader and more heterogeneous size distributions, likely due to the complex and variable nature of biological reducing and stabilizing agents.

The TEM histogram analysis of CSN obtained under optimal conditions reveals the smallest average diameter (2.11 ± 0.07 nm), approximately 20 times smaller than the average size of the biosynthesized samples (Table 2). This pronounced size reduction is advantageous, as smaller nanoparticles possess a higher surface-area-to-volume ratio, which can enhance their chemical reactivity and antimicrobial performance.

**Table 2:** Nanoparticle Diameter (TEM) and Silver Content (TXRF) of Chemosynthesized (CSN) and Biosynthesized (BSN1, BSN2, and BSN3) Silver Nanoparticle Dispersion^a^

Silver nanoparticles obtained by	Diameter of silver nanoparticles (nm)	Silver content in the nanoparticle dispersion (mg L^–1^)
**Chemosynthesis (CSN)**	2.11 ± 0.07 a	614.03 ± 2.29 A
**Biosynthesis (BSN1)**	32.4 ± 4.7 b	571.63 ± 19.15 B
**Biosynthesis (BSN2)**	49.5 ± 4.8 c	406.94 ± 4.88 C
**Biosynthesis (BSN3)**	45.5 ± 9.6 bc	584.35 ± 22.70 B

a BSN1, BSN2, and BSN3 refer to silver nanoparticles biosynthesized using Aspergillus niger spore suspensions of 1 mL (6.4 × 106 spores), 1.5 mL (9.6 × 106 spores), and 2 mL (12.8 × 106 spores), respectively, cultivated in 100 mL glucose-casein hydrolyzate liquid medium. Values are expressed as Gaussian mean ± standard deviation derived from histogram fits in Figure 4. Different uppercase or lowercase letters within the same column denote statistically significant differences according to Student’s t test (p ≤ 0.05).

In addition to particle size and morphology, the elemental composition of the nanoparticle dispersions was assessed by TXRF (Table 3). As expected, silver was the predominant element in all treatments, in agreement with the previous TEM–TXRF results (Table 2). Beyond silver, trace elements such as Al, Fe, Cu, and Zn were detected at variable levels depending on the synthesis method. Biosynthesized samples exhibited higher aluminum concentrations than CSN, likely associated with fungal metabolites or medium components, whereas CSN showed comparatively greater zinc content. Gallium, used as the internal standard, remained consistent across all samples, with minor variations (≤4%) considered normal for TXRF analyses. Other elements (Cr, Mn, Co, As, Rb, Sr, Mo, and Pb) were also screened but were not detected within the method’s detection limits. Overall, these results show that although chemical and biological syntheses introduce minor elemental impurities characteristic of their respective origins, such traces do not compromise the overall composition, which remains clearly dominated by silver nanoparticles.

**Table 3:** Concentrations of Chemical Elements in Chemosynthesized (CSN) and Biosynthesized (BSN1, BSN2, and BSN3) Silver Nanoparticle Samples, Determined by Total Reflection X-ray Fluorescence (TXRF)^a^

Element	CSN	BSN1	BSN2	BSN3
	mg L^–1^			
**Al**	0.24 ± 0.07 d	0.62 ± 0.06 c	0.84 ± 0.05 b	0.97 ± 0.04 a
**Fe**	0.24 ± 0.04 a	0.09 ± 0.04 b	0.16 ± 0.05 b	0.17 ± 0.05 b
**Ni**	0.07 ± 0.01 a	0.08 ± 0.03 a	0.08 ± 0.01 a	0.08 ± 0.01 a
**Cu**	0.13 ± 0.01 a	0.08 ± 0.01 b	0.07 ± 0.01 b	0.07 ± 0.01 b
**Zn**	0.38 ± 0.01 a	0.22 ± 0.01 b	0.17 ± 0.01 c	0.16 ± 0.02 c
**Au**	–	0.11 ± 0.01 a	0.07 ± 0.02 a	0.10 ± 0.02 a
**Ag**	614.03 ± 2.29 a	571.63 ± 19.15 b	406.94 ± 4.88 c	584.35 ± 22.70 b
**Ga**	100.35 ± 0.09 c	102.02 ± 0.09 b	100.12 ± 0.09 c	103.87 ± 0.10 a

a BSN1, BSN2, and BSN3 refer to silver nanoparticles biosynthesized using Aspergillus niger spore suspensions of 1 mL (6.4 × 106 spores), 1.5 mL (9.6 × 106 spores), and 2 mL (12.8 × 106 spores), respectively, cultivated in 100 mL glucose-casein hydrolyzate liquid medium. Ga = gallium, used as the internal standard. Different letters within the same row denote statistically significant differences according to Student’s t test (p ≤ 0.05). Other elements (Cr, Mn, Co, As, Rb, Sr, Mo, and Pb) were analyzed but not detected within the method’s detection limits. – = indicates elements below the limit of detection.

Although biosynthesis produced larger particles, it offered distinct benefits, including environmental sustainability, reduced toxicity, and reliance on renewable biological resources. Among the biosynthesized groups, BSN1, obtained with 1 mL spore suspension, produced the smallest nanoparticles (32.4 ± 4.7 nm, Table 2). BSN3, obtained with 2 mL spore suspension, yielded intermediate-sized particles (45.5 ± 9.6 nm), whereas BSN2, obtained with 1.5 mL spores, resulted in the largest nanoparticles (49.5 ± 4.8 nm, Table 2). These results suggest that lower spore concentrations, particularly 1 mL, may favor the formation of smaller nanoparticles in biosynthesis, as observed in BSN1 (Table 2).

To complement the average diameters and standard deviations, fwhm values were calculated from Gaussian fits applied to the TEM-based size distribution histograms (Figure 4). The fwhm of CSN was 0.97 nm, confirming its high monodispersity. In contrast, the biosynthesized samples exhibited broader distributions: 33.0 nm (BSN1), 28.4 nm (BSN2), and 48.5 nm (BSN3). These results reinforce that CSN had the narrowest and most uniform size distribution, whereas the BSNs showed greater polydispersity. However, BSN2 stood out for having a narrower distribution than BSN1 and BSN3, indicating relatively improved homogeneity, despite yielding the largest particles.

CSN exhibited the highest silver content in solution (614.03 ± 2.29 mg L^–1^), significantly surpassing all biosynthesized samples (Table 2). Among the biosynthesized samples, BSN3 and BSN1 showed comparably high silver contents—584.35 ± 22.70 mg L^–1^ and 571.63 ± 19.15 mg L^–1^, respectively—while BSN2 presented the lowest content (406.94 ± 4.88 mg L^–1^) (Table 2). These results indicate that lower spore concentrations, such as 1 mL, can be as effective as the higher spore concentration (2 mL) in maximizing nanoparticle yield under biosynthetic conditions. This highlights that the silver content in CSN was approximately 1.2 times greater than the average content of the biosynthesized samples, underscoring the higher efficiency of the chemical synthesis method. Furthermore, the chemical synthesis was repeated in three independent batches, all of which yielded nanoparticles with mean diameters close to 2 nm and narrow size distributions, as confirmed by TEM. The consistency in particle size and morphology across replicates attests to the robustness and reliability of the optimized chemical protocol, demonstrating its ability to consistently produce monodisperse and well-defined silver nanoparticles under controlled conditions.

### 3.4 Antibacterial Activity

CSN and BSNs exhibited bacteriostatic activity against *C. perfringens*, *E. coli*, *P. aeruginosa*, and *S. aureus* (Table 4). CSN demonstrated the highest efficacy among all treatments, presenting the lowest MIC values across all tested strains (Table 4). CSN achieved MIC values of 19.19 μg mL^–1^ against *C. perfringens* and *E. coli* and 38.38 μg mL^–1^ against *P. aeruginosa* and *S. aureus* (Table 4). Although CSN was less potent than the reference antibiotic ampicillin against susceptible strains, it effectively inhibited *P. aeruginosa*, which is resistant to ampicillin, highlighting its broad-spectrum antimicrobial potential.

**Table 4:** Minimum Inhibitory Concentration (MIC) Values for the Bacteriostatic Activity of Chemosynthesized Silver Nanoparticles (CSN), Biosynthesized Silver Nanoparticles Using *Aspergillus niger* (BSN1, BSN2, BSN3), and the Positive Control Antibiotic Ampicillin against Selected Bacterial Strains^a^

	MIC (μg mL^–1^)				
Bacterium	CSN	BSN1	BSN2	BSN3	Ampicillin
*Clostridium perfringens*	19.19	142.91	50.87	36.52	1.25
*Escherichia coli*	19.19	71.45	50.87	146.09	1.56
*Pseudomonas aeruginosa*	38.38	71.45	50.87	73.04	Resistant
*Staphylococcus aureus*	38.38	71.45	50.87	73.04	0.97

a BSN1, BSN2, and BSN3 refer to silver nanoparticles biosynthesized using Aspergillus niger spore suspensions of 1 mL (6.4 × 106 spores), 1.5 mL (9.6 × 106 spores), and 2 mL (12.8 × 106 spores), respectively, cultivated in 100 mL glucose-casein hydrolyzate liquid medium. MIC values (*n* = 3) are reported as consensus thresholds, following CLSI guidelines,^18^ which do not require standard deviation reporting.

Ampicillin showed the most significant overall potency, with MIC values of 0.97 μg mL^–1^ for *S. aureus*, 1.25 μg mL^–1^ for *C. perfringens*, and 1.56 μg mL^–1^ for *E. coli*, but fails to inhibit *P. aeruginosa* (Table 4). Compared to ampicillin, CSN is approximately 15 times less potent against *C. perfringens*, 12 times less potent against *E. coli*, and 40 times less potent against *S. aureus*.

BSNs were obtained using increasing concentrations of *A. niger* spores—BSN1 (1 mL), BSN2 (1.5 mL), and BSN3 (2 mL)—and a general trend of enhanced bacteriostatic activity was observed with greater fungal biomass. BSN1 exhibited the weakest performance, with high MIC values across all strains (Table 4). BSN2 showed the most consistent and effective results, achieving MIC values of 50.87 μg mL^–1^ for all tested bacteria (Table 4). BSN3, although synthesized with the highest spore concentration, exhibited variable efficacy, showing the lowest MIC value among the BSNs against *C. perfringens* (36.52 μg mL^–1^), indicating the highest potency for this strain (Table 4). However, it displayed reduced activity against *E. coli* (146.09 μg mL^–1^), *P. aeruginosa* (73.04 μg mL^–1^), and *S. aureus* (73.04 μg mL^–1^), underperforming compared to BSN2 for these bacteria (Table 4).

When comparing the most effective BSNs to CSN and ampicillin, BSN3 exhibits a MIC value against *C. perfringens* that was 29 times higher than ampicillin and 1.9 times higher than CSN, indicating lower potency. Similarly, BSN2 required MIC values 33 times higher than ampicillin to inhibit *E. coli* and 52 times higher to inhibit *S. aureus*, reflecting reduced potency. While most BSNs were effective, BSN2, in particular, inhibited *P. aeruginosa* with a MIC value only 1.3 times higher than that of CSN. Among the BSN samples, BSN2 exhibited the most consistent and overall better bacteriostatic performance across the tested strains, except for *C. perfringens*, where BSN3 proved more effective. This highlights their potential as alternative agents for combating antibiotic-resistant bacteria.

Overall, CSN exhibited superior bacteriostatic efficacy, while BSN2 stood out as the most promising among the biosynthesized variants. Despite lower potency than CSN and ampicillin, BSNs remained effective against resistant bacteria. These findings suggest that biosynthetic approaches, particularly fungal-mediated synthesis, play a critical role in determining nanoparticle performance and can be optimized to enhance antimicrobial activity.

The MBC values revealed distinct differences in antimicrobial efficacy among silver nanoparticle treatments synthesized via chemical and biological methods (Table 5). CSN consistently demonstrated the most potent bactericidal activity across all tested bacterial strains (Table 5). CSN achieved a MBC value of 19.19 μg mL^–1^ against *C. perfringens* and 38.38 μg mL^–1^ against *E. coli*, *S. aureus*, and *P. aeruginosa* (Table 5). While ampicillin exhibited greater potency against *C. perfringens* (1.47 μg mL^–1^), *E. coli* (2.43 μg mL^–1^), and *S. aureus* (1.14 μg mL^–1^), it was ineffective against *P. aeruginosa*, confirming the strain’s known resistance profile (Table 5). Additionally, CSN effectively eliminated *P. aeruginosa*, indicating its potential as an alternative antimicrobial agent for treating resistant infections.

**Table 5:** Minimum Bactericidal Concentration (MBC) Values for the Bactericidal Activity of Chemosynthesized Silver Nanoparticles (CSN), Biosynthesized Silver Nanoparticles Using *Aspergillus niger* (BSN1, BSN2, BSN3), and the Positive Control Antibiotic Ampicillin Against Selected Bacterial Strains^a^

	MBC (μg mL^–1^)				
Bacterium	CSN	BSN1	BSN2	BSN3	Ampicillin
*Clostridium perfringens*	19.19	142.91	50.87	73.04	1.47
*Escherichia coli*	38.38	142.91	101.74	146.09	2.43
*Pseudomonas aeruginosa*	38.38	71.45	50.87	73.04	Resistant
*Staphylococcus aureus*	38.38	71.45	50.87	73.04	1.14

a BSN1, BSN2, and BSN3 refer to silver nanoparticles biosynthesized using Aspergillus niger spore suspensions of 1 mL (6.4 × 106 spores), 1.5 mL (9.6 × 106 spores), and 2 mL (12.8 × 106 spores), respectively, cultivated in 100 mL glucose-casein hydrolyzate liquid medium. MBC values (n = 3) are reported as consensus thresholds, following CLSI guidelines,^18^ which do not require standard deviation reporting.

Compared to ampicillin, the CSN MBC values were approximately 13 times higher for *C. perfringens*, 16 times higher for *E. coli*, and 34 times higher for *S. aureus*. Although less potent than ampicillin for susceptible strains, CSN’s ability to inhibit *P. aeruginosa* highlights its clinical relevance in addressing antibiotic-resistant bacteria.

Among BSNs, BSN2—produced with 1.5 mL spores—demonstrated the most consistent and effective bactericidal performance. BSN2 exhibited MBC values of 50.87 μg mL^–1^ against *C. perfringens*, *P. aeruginosa*, and *S. aureus* and 101.74 μg mL^–1^ against *E. coli* (Table 5). Compared to ampicillin, BSN2’s MBC values were 35 times higher for *C. perfringens*, 42 times higher for *E. coli*, and 45 times higher for *S. aureus*, indicating lower relative potency.

BSN1, produced with 1 mL spores, the lowest spore concentration, exhibited the weakest bactericidal activity, with MBC values of 142.91 μg mL^–1^ for *C. perfringens* and *E. coli* and 71.45 μg mL^–1^ for *P. aeruginosa* and *S. aureus* (Table 5). BSN3, produced with 2 mL spores, the highest spore concentration, exhibited a mixed performance, showing greater efficacy than BSN1 against *C. perfringens* (73.04 μg mL^–1^) but similar activity against the other strains and consistently lower efficacy than BSN2.

When directly comparing BSN2 to CSN, the BSN2MBC values were approximately 2.6 times higher for *C. perfringens* and *E. coli* and 1.3 times higher for *P. aeruginosa* and *S. aureus*, confirming CSN’s overall superior efficacy. Nonetheless, BSNs—particularly BSN2—remained functionally active and relevant, mainly due to their ability to inhibit *P. aeruginosa*, which is resistant to conventional antibiotics.

Despite BSN2 exhibiting the largest average diameter of silver nanoparticles (49.5 ± 4.8 nm) and the lowest silver content among the biosynthesized samples (406.94 mg L^–1^) (Table 2), it demonstrated the most effective antibacterial activity against all tested bacterial strains within the BSN treatments (Table 5). This superior performance, seemingly counterintuitive given its physicochemical profile, likely resulted from synergistic factors that transcend particle size and silver content alone. Moreover, nanoparticle morphology, surface characteristics, and colloidal stability can significantly affect antimicrobial efficacy. BSN2 nanoparticles may present enhanced dispersion or surface reactivity, facilitating interaction with bacterial membranes. Additionally, the biosynthesis process using *A. niger* could have led to the capping of nanoparticles with bioactive fungal metabolites, which are known to exert antimicrobial effects and potentially enhance the delivery or retention of silver ions at the site of action. These surface-bound compounds may also promote a more sustained and bioavailable release of silver ions, compensating for the lower overall silver content. Furthermore, subtle differences in biosynthesis conditions—such as pH, precursor concentration, or fungal biomass—might have resulted in nanoparticles with distinct biochemical and functional properties. An additional explanation for the superior antibacterial activity of BSN2 lies in its bimodal size distribution. Unlike BSN1 and BSN3, BSN2 contains both smaller (<10 nm) and larger (>20 nm) nanoparticles, which may act synergistically (Figure 4C). Smaller particles are more effective at penetrating bacterial membranes, while larger ones can ensure sustained silver ion release. This dual-size profile may enhance overall antibacterial efficacy, even with a lower silver content. Although no fractionation or mechanistic analysis was conducted to isolate the specific contributions of each size group, this hypothesis is consistent with previously reported synergistic effects in mixed-size silver nanoparticle systems.^7,8,13^ Further studies employing size-resolved antimicrobial assays and nanoparticle-cell interaction analyses would be valuable to confirm this proposed mechanism. These findings underscore that antimicrobial efficacy is governed not solely by nanoparticle size or silver content but by a complex interplay of physicochemical and biological parameters.

Overall, chemical and biological synthesis methods produced silver nanoparticles with significant bactericidal activity. CSN consistently outperformed BSNs in potency, but BSN2 stood out among the biosynthesized variants, suggesting that an intermediate spore concentration may have favored optimal nanoparticle properties. The effective inhibition of *P. aeruginosa* by CSN and BSNs reinforced the potential of silver nanoparticles as alternative antimicrobial agents in combating drug-resistant bacterial infections.

## 4 Discussion

Chemical synthesis produced silver nanoparticles with smaller particle sizes and greater conversion efficiency of silver ions compared to fungal-mediated biological synthesis (BSN) treatments. Under optimized conditions (0.7 g L^–1^ CMC, 6.0 × 10^–3^ mol L^–1^ NaBH_4_, and 3.5 × 10^–4^ mol L^–1^ silver nitrate), CSN demonstrated more efficient and controlled nanoparticle production. In contrast, BSN using *A. niger* generated particles up to 22 times larger in diameter; however, silver recovery remained comparable—particularly in BSN1, which achieved a favorable trade-off between particle size and silver content. Moreover, the biosynthesis process exhibited a sublinear growth trend relative to inoculum concentration, primarily limited by the culture medium’s capacity, which aligns with the characteristics of batch processing.

UV–vis spectroscopy confirmed the presence of stable, well-dispersed silver nanoparticles in CSN and BSN samples, with CSN showing sharp and well-defined surface plasmon resonance peaks. TEM analysis revealed that CSN generates spherical nanoparticles with narrow size distributions and minimal agglomeration. In contrast, BSNs showed greater morphological diversity and broader size distributions, which were attributed to variations in spore inoculum and the complexity of the biological medium.

Using toxic reagents in CSN, such as sodium borohydride, raises concerns regarding environmental safety and human health. Although no experimental analysis of degradation or silver ion release was conducted in our study, the CSN may exhibit greater persistence—both colloidal and environmental—compared to biosynthesized counterparts. This inference is based on their narrow size distribution, spherical morphology, and especially the presence of CMC as a synthetic polymeric stabilizer, which is known to reduce biodegradability and enhance long-term stability.^6^ In contrast, BSNs are capped with fungal biomolecules that are potentially more prone to enzymatic or microbial degradation. While this remains a plausible hypothesis grounded in physicochemical characteristics and literature precedent, it was not experimentally verified and should be further evaluated in future studies.

In contrast, BSN employs natural biomolecules that form organic capping layers, promoting a more controlled release of silver ions and reducing environmental toxicity.^8,28^ The greener chemistry underlying BSN offers enhanced ecological compatibility, making it a more sustainable choice, especially for biomedical and environmental applications.

CSN and BSN treatments exhibited bacteriostatic and bactericidal activity against all tested strains, including *C. perfringens*, *E. coli*, *S. aureus*, and ampicillin-resistant *P. aeruginosa*. CSN consistently showed superior efficacy, with lower MIC and MBC values across most strains. Among BSNs, BSN2 was the most effective in controlling all bacterial strains. The antimicrobial action correlated with nanoparticle size, stability, and ion release rather than silver content alone, reaffirming the role of structural-functional properties in biological performance.

*C. perfringens* is a Gram-positive, spore-forming anaerobe found in soil, food, sewage, and the gut microbiota of humans and animals, and can cause diseases such as gas gangrene, food poisoning, and enterocolitis.^29^ In our study, silver nanoparticles synthesized via chemical methods (CSN) exhibited MIC and MBC values of 19.19 μg mL^–1^ (2.11 ± 0.07 nm particle size) against *C. perfringens*. In contrast, the BSNs, more specifically BSN3, showed a MIC of 36.52 μg mL^–1^ (45.5 ± 9.6 nm particle size), while BSN2 demonstrated an MBC of 50.87 μg mL^–1^ (49.5 ± 4.8 nm particle size). These results align with previously reported MIC values for silver nanoparticles against *C. perfringens*, which range from 20 μg mL−1 ^30^ to 100 μg mL^–1^,^31^ confirming the consistency and reliability of our findings.

For *E. coli*, a Gram-negative gut commensal, the emergence of antibiotic-resistant and toxigenic strains has turned it into a significant clinical threat, especially in immunocompromised individuals, highlighting the need for effective control strategies.^32^ In our study, silver nanoparticles synthesized via chemical methods (CSN) achieved MIC and MBC values of 19.19 μg mL^–1^ and 38.38 μg mL^–1^, respectively, while BSN2 showed MIC of 50.87 μg mL^–1^ and MBC of 101.74 μg mL^–1^ against *E. coli*. These MIC values are comparable to those previously reported: 8 μg mL^–1^,^7^ 11.25–22.5 μg mL^–1^ for 1.9–6.5 nm particles,^1^ 30 μg mL^–1^ for 5–10 nm particles,^3^ and 75 μg mL^–1^ for BSN synthesized via *Aspergillus flavus*.^33^ Similarly, previously reported MBC values—32 μg mL−1 ^7^ and 40 μg mL−1 ^3^ —are consistent with our study’s observations.

For *P. aeruginosa*, a clinically important Gram-negative bacterium, pathogenicity is driven by environmental adaptability, biofilm formation, and multidrug resistance, making antibiotic resistance a major treatment challenge.^34^ In our study, silver nanoparticles synthesized via CSN and BSN2 exhibited MIC and MBC values of 38.38 μg mL^–1^ and 50.87 μg mL^–1^, respectively, against *P. aeruginosa*. Previously reported MIC values for *P. aeruginosa* range from 5.65–11.25 μg mL^–1^,^1,7^ while multidrug-resistant *P. aeruginosa* strains have shown MICs of 1.406–5.625 μg mL^–1^ and MBCs of 2.813–5.625 μg mL−1 ^13^ and 64 μg mL^–1^.^7^ Despite our study’s higher MIC and MBC values, the performance of BSN2 remains within a comparable range to values previously reported in the literature.

For *S. aureus*, a Gram-positive bacterium causing infections from mild to severe, its clinical relevance is amplified by its role in healthcare-associated infections and the rise of methicillin-resistant strains (MRSA), posing a primary global health concern.^35^ In our study, silver nanoparticles synthesized via CSN exhibited MIC and MBC values of 38.38 μg mL^–1^, while BSN2 showed MIC and MBC values of 50.87 μg mL^–1^. Reported MIC values for *S. aureus* vary widely, from 2.5–25 μg mL^–1^ ^14^ to 80 μg mL^–1^ ^3^ and 128 μg mL^–1^,^7^ with MRSA strains ranging from 22.5 to 45 μg mL^–1^.^1^ Previously reported MBC values span from 2.813–5.625 μg mL^–1^ ^13^ to 100 μg mL^–1^ ^3^ and up to 256 μg mL^–1^.^7^ These findings indicate that the MIC values in our study are consistent with the literature, while MBC values fall within the broad variability reported. Although BSN2 required a slightly higher concentration than CSN to control *S. aureus*, both values fall within the expected efficacy range for silver nanoparticles and reflect complete bacterial suppression under the tested conditions. Thus, while CSN may be considered more potent, as it achieves inhibition at a lower concentration, CSN and BSN2 are equally effective antimicrobials within the context of the experimental design and concentration intervals tested.

Reported MBC values for *S. aureus* vary widely in the literature, ranging from as low as 2.81 μg mL^–1^ to over 250 μg mL^–1^.^7,13^ This broad variability is largely attributed to differences in the physicochemical properties of silver nanoparticles, including size, shape, dispersion, and surface chemistry, all of which are strongly affected by the synthesis method employed.^5,9^ Smaller, spherical, and more monodisperse nanoparticles typically exhibit higher antibacterial potency due to their increased surface area and enhanced ability to interact with bacterial membranes.^1,3^ Conversely, larger or polydisperse particles may release silver ions more slowly or less uniformly.^5,7^ Additionally, the presence of specific capping agents—whether synthetic polymers or biologically derived metabolites—can modulate nanoparticle charge and stability, further impacting antimicrobial efficacy.^6,8^ In our study, the observed MBC values fall within this reported range and are consistent with the expected biological performance based on the nanoparticles’ size, morphology, and synthesis approach. These factors help explain the antibacterial outcomes and reinforce the need for careful nanoparticle characterization in antimicrobial research.

Furthermore, Haidari et al.^1^ reported over 70% cell viability in human fibroblasts exposed to silver nanoparticles at a concentration of 90  μg mL^–1^, indicating that the concentrations used in our study—except for the highest MBC observed among the biosynthesized nanoparticles—remain within acceptable biocompatibility thresholds. This supports the potential applicability of silver nanoparticles, particularly those biosynthesized, in medical contexts. Our findings confirm that although CSN provides superior efficiency, yield, and antibacterial activity, it raises critical environmental sustainability and safety concerns. In contrast, BSN, especially when employing *A. niger*, offers a greener and safer alternative, demonstrating promising antimicrobial performance despite larger particle sizes and slightly lower potency. These results highlight the importance of tailoring synthesis methods to specific application demands—favoring CSN for industrial-scale efficiency and BSN for environmentally friendly biomedical uses. Future research should focus on optimizing BSN protocols to enhance nanoparticle uniformity and functional efficacy without compromising their sustainable nature.

While the BSNs demonstrated promising antimicrobial activity and present potential for biomedical applications, their use in clinical settings faces important scalability and regulatory challenges.^8,28^ Biosynthesis using a fungus involves biological complexity that can result in batch-to-batch variability, requiring stringent process control to ensure reproducibility in particle size, morphology, and purity.^9,12^ Moreover, the presence of residual fungal biomolecules—although possibly contributing to biological activity—raises concerns regarding sterility, immunogenicity, and potential toxicity.^6,8^ Regulatory agencies demand rigorous validation of nanoparticle safety, biocompatibility, and stability, as well as standardized protocols for purification and characterization.^28^ While CSN may be more straightforward to scale under Good Manufacturing Practice (GMP) conditions, BSNs will require further investigation and process optimization before clinical translation becomes feasible.^8,28^ Nonetheless, the inherent biocompatibility and green chemistry approach of BSNs remain attractive features that justify continued exploration.

The potential of BSNs to combat antibiotic-resistant bacteria lies in their multimodal mechanisms of action, which differ fundamentally from conventional antibiotics. Unlike β-lactams or other antibiotics that target specific cellular components, silver nanoparticles exert their antimicrobial effects through a combination of membrane disruption, oxidative stress induction, and interference with DNA and protein functions.^5,7,8^ These nonspecific mechanisms reduce the likelihood of resistance development and may allow silver nanoparticles to retain activity even against multidrug-resistant organisms, such as methicillin-resistant *S. aureus* (MRSA) or carbapenem-resistant *P. aeruginosa*.^1,13^ In our study, although the *S. aureus* strain used is not methicillin-resistant, the observed bactericidal activity of BSNs suggests that these nanoparticles could be effective against resistant strains, given their broad-spectrum and nonselective mode of action. However, several limitations must be considered. First, the heterogeneity of BSNs in terms of size, shape, and surface chemistry—arising from the complex biological synthesis process—can lead to variability in antimicrobial performance and poses challenges for clinical standardization.^8,9,12^ Second, the presence of biomolecular capping agents from fungal origin, while contributing to nanoparticle stability and possibly enhancing antimicrobial action, may also raise concerns regarding biocompatibility, immunogenicity, and batch-to-batch reproducibility.^6,8,28^ Third, limited data exist on the pharmacokinetics, biodistribution, and long-term safety of BSNs *in vivo*, particularly in the context of systemic infections caused by resistant pathogens.^8,28^ Therefore, while BSNs offer a promising eco-friendly and potentially effective strategy for addressing the growing threat of antimicrobial resistance, their translation into clinical use against resistant strains such as MRSA or extended-spectrum β-lactamase (ESBL)-producing bacteria will require further optimization, rigorous standardization, and comprehensive preclinical evaluation.^8,28^ Nonetheless, their demonstrated efficacy against ampicillin-resistant *P. aeruginosa* in our study reinforces their relevance as candidates for continued research in antimicrobial nanotechnology.

Across *E. coli*, *S. aureus*, *C. perfringens*, and *P. aeruginosa*, CSN consistently demonstrated lower MIC and MBC values compared with BSN. For instance, in *E. coli*, CSN achieved a MIC of 19.19 μg mL^–1^ and an MBC of 38.38 μg mL^–1^, whereas BSN2 required 50.87 μg mL^–1^ and 101.74 μg mL^–1^, respectively. Similarly, in *S. aureus*, both the MIC and MBC for CSN were 38.38 μg mL^–1^, while BSN2 required 50.87 μg mL^–1^ for both end points. These results support the established relationship between reduced particle size, controlled surface properties, and enhanced antimicrobial potency.^3,36^

Size-controlled studies show that MIC and MBC decrease as particle diameter becomes smaller, with silver nanoparticles below 10 nm generally exhibiting the highest activity.^3^ In our results, the MIC for *E. coli* (19.19 μg mL^–1^) falls within or below the reported range of 20–110 μg mL^–1^, while the MIC for *S. aureus* (38.38 μg mL^–1^) is within the reported 70–200 μg mL^–1^ range. In the sub-10 nm domain, particles around 3 nm enriched in surface monovalent silver achieve the lowest MIC values (5.65–22.5 μg mL^–1^) and maintain favorable therapeutic windows. This highlights the superior potency of ultrasmall, monovalent silver-accessible surfaces—consistent with the enhanced performance of our ∼2 nm CSN compared with the larger BSN.^1^

Across the four indicator organisms, our MIC and MBC values were generally consistent with ranges reported in the literature when accounting for differences in particle size, capping agents, and assay conditions. For *C. perfringens*, CSN exhibited a MIC of 19.19 μg mL^–1^ (2.11 ± 0.07 nm), while BSN3 and BSN2 required 36.52 μg mL^–1^ (45.5 ± 9.6 nm) and 50.87 μg mL^–1^ (49.5 ± 4.8 nm), respectively. These results align with reported MIC values of approximately 20–100 μg mL^–1^.^30,31^ For *E. coli*, CSN achieved a MIC of 19.19 μg mL^–1^ and an MBC of 38.38 μg mL^–1^, whereas BSN2 required 50.87 μg mL^–1^ and 101.74 μg mL^–1^, respectively. These values are comparable to previous reports of 8 μg mL^–1^,^7^ 11.25–22.5 μg mL^–1^ for particles of 1.9–6.5 nm,^1^ ∼30 μg mL^–1^ for 5–10 nm particles,^3^ and 75 μg mL^–1^ for *A. flavus*-derived silver nanoparticles,^33^ as well as MBC values of 32–40 μg mL^–1^.^3,7^ For *P. aeruginosa*, the MIC was 38.38 μg mL^–1^ and the MBC was 50.87 μg mL^–1^. Although these values are higher than some optimized sub-10 nm systems (MIC 5.65–11.25 μg mL^–1^),^1,7^ they remain within the range of other studies, including multidrug-resistant strains (MIC 1.406–5.625 μg mL^–1^; MBC 2.813–5.625 μg mL^–1^)^13^ and reports up to 64 μg mL^–1^.^7^ For *S. aureus*, CSN reached both a MIC and MBC of 38.38 μg mL^–1^, while BSN2 required 50.87 μg mL^–1^ for both. These values fall within reported ranges spanning MICs of 2.5–128 μg mL^–1^,^3,7,14^ and MBCs of 2.813–256 μg mL^–1^.^3,7,13^ Overall, these comparisons confirm that antimicrobial potency scales with key physicochemical factors—namely, smaller particle size, narrower dispersity, and favorable surface states—while variations across studies are likely attributable to differences in nanoparticle characteristics and assay protocols.^1,3,5,6,8^

Silver nanoparticles exert antimicrobial effects through multiple pathways, including membrane disruption, generation of reactive oxygen species, and interference with DNA and protein synthesis, in addition to the release of silver ions. These combined mechanisms underpin their broad activity against both Gram-positive and Gram-negative bacteria, as well as multidrug-resistant strains.^37,38^ In multidrug-resistant *P. aeruginosa*, reported MIC and MBC values range from approximately 1.4 to 5.6 μg mL^–1^, accompanied by evidence of reactive oxygen species overproduction and apoptosis-like cellular responses.^13^ Reviews consistently emphasize the critical role of particle size, surface state, and silver ion availability as the principal determinants of antimicrobial potency.^6,37,39^

Chemical reduction methods (e.g., borohydride or citrate) offer precise and reproducible control over nanoparticle synthesis but rely on hazardous reagents.^28^ In contrast, green or biogenic approaches—using plants, microbes, fungi, lichens, or agricultural wastes—replace harsh chemicals with endogenous reducing and capping agents. These methods often produce biocompatible surface coatings but typically result in broader particle size distributions and greater batch-to-batch variability, which is consistent with the higher potency observed for CSN compared with BSN.^6–8,37,40–42^ Among biogenic routes, fungal systems are particularly appealing because of enzyme-mediated reduction and stabilization, as well as the potential for synergistic effects with fungal-derived antibiotics.^33^ However, they face practical barriers to large-scale application, including challenges in sterility, immunogenicity, and standardization under regulatory requirements.^6,8,28^

Quantitative and standardized MIC and MBC protocols, coupled with transparent reporting of dose, exposure, and assay format, are essential for improving comparability across studies. Inhibition zone measurements generally scale with dose and are relatively unaffected by storage conditions.^43^ Reported applications include surface coatings, medical devices, and drug-delivery systems, where silver nanoparticles can enhance therapeutic efficacy while reducing off-target toxicity.^38,44^ Cell-compatibility studies indicate over 70% fibroblast viability at concentrations up to 90 μg mL^–1^,^1^ supporting the biomedical relevance of our tested dose ranges, except for the highest MBC recorded for the biosynthesized nanoparticles.

To reduce the performance gap between CSN and BSN without compromising sustainability, several strategies can be considered: (i) directing BSN synthesis toward sub-10 nm cores with narrower size distributions by adjusting green-synthesis variables such as metal-to-biomass ratio, pH, temperature, and reaction time; (ii) selecting capping agents that ensure colloidal stability while allowing controlled release of silver ions; and (iii) standardizing MIC/MBC protocols and bacterial strain panels.^45^ Additional shape control to favor high-surface-area facets may further decrease effective antimicrobial doses.^14^ In our study, ∼ 2 nm monodisperse CSN achieved MIC and MBC values comparable to the best-performing sub-10 nm systems reported in the literature and consistently outperformed BSN with sizes of 45–50 nm. These results highlight that particle size, dispersity, and surface state—including accessible silver ion—are decisive determinants of potency and can be rationally engineered within greener synthesis workflows.^1,3,6,8,28,36,40^

## 5 Conclusions

This study demonstrates that the effectiveness of CSN production depends on optimizing the concentrations of sodium borohydride and carboxymethylcellulose used in the synthesis process. The optimal condition—6.0 × 10^–3^ mol L^–1^ NaBH_4_ and 0.7 g L^–1^ CMC—maximizes the analytical response under a fixed silver nitrate concentration of 3.5 × 10^–4^ mol L^–1^. The results confirm that a balanced interaction between reducing and stabilizing agents is essential for efficient nanoparticle formation.

Fungal-mediated biosynthesis of silver nanoparticles shows that increasing the spore inoculum enhances fungal biomass production but with diminishing efficiency. The nonlinear growth trend suggests environmental limitations in the culture medium. All treatments produce silver nanoparticles, with color variations indicating differences in synthesis dynamics that may affect nanoparticle characteristics.

UV–vis analysis confirms that chemical and biological routes produce stable, spherical silver nanoparticles with minimal aggregation and high monodispersity. The optimized chemical method produces nanoparticles with sharp, well-defined surface plasmon resonance peaks, whereas increasing fungal biomass in biosynthesis leads to greater size and shape diversity. The spectral similarity between BSN3 and CSN demonstrates that, under optimized conditions, biosynthesis can achieve physicochemical characteristics comparable to those obtained via chemical synthesis.

TEM analysis reveals that CSN exhibits a uniform spherical morphology, narrow size distribution, and minimal agglomeration, reflecting controlled synthesis and effective polymeric stabilization. In contrast, BSNs show greater morphological variation, broader size distributions, and increased aggregation, affected by *A. niger* spore concentration. These results highlight the superior structural uniformity of CSN and the biological complexity and tunability of BSN synthesis.

Chemosynthesis produces silver nanoparticles with significantly smaller sizes and higher concentrations than biosynthesis, demonstrating superior efficiency and control. However, biosynthetic methods using *A. niger* provide environmentally sustainable alternatives. BSN1 (1 mL spore suspension) yields the smallest biosynthesized particles with a relatively high silver concentration, indicating that lower spore levels can optimize nanoparticle features. Nonetheless, chemosynthesis remains the most effective method for achieving uniformity and maximum yield.

Silver nanoparticles synthesized via both methods exhibit bacteriostatic and bactericidal activity against *C. perfringens*, *E. coli*, *S. aureus*, and ampicillin-resistant *P. aeruginosa*. CSN consistently achieves the highest overall efficacy, outperforming all biosynthesized treatments. Among the BSNs, BSN2 delivers the most potent and consistent antibacterial effect. Although BSNs—particularly BSN2—demonstrate lower potency than CSN and ampicillin, they represent a promising alternative for addressing antibiotic-resistant infections. The evidence suggests that antimicrobial efficacy arises from a complex interplay of structural and functional properties rather than silver content alone. This highlights the potential applications of sustainably synthesized silver nanoparticles in food packaging, healthcare, and environmental protection—particularly for combating ampicillin-resistant *P. aeruginosa*.
